# Complete Genome Sequence of Cell Culture-Attenuated Guinea Pig Cytomegalovirus Cloned as an Infectious Bacterial Artificial Chromosome

**DOI:** 10.1128/genomeA.00928-14

**Published:** 2014-10-16

**Authors:** Dongmei Yang, Zohaib Alam, Xiaohong Cui, Michael Chen, Carly J. Sherrod, Michael A. McVoy, Mark R. Schleiss, Dirk P. Dittmer

**Affiliations:** a University of North Carolina School of Medicine, Lineberger Comprehensive Cancer Center and Department of Microbiology and Immunology, Chapel Hill, North Carolina, USA; b Virginia Commonwealth University School of Medicine, Department of Pediatrics, Richmond, Virginia, USA; c University of Minnesota Medical School, Department of Pediatrics, Division of Pediatric Infectious Diseases and Immunology, Minneapolis, Minnesota, USA

## Abstract

The complete genome sequence of attenuated guinea pig cytomegalovirus cloned as bacterial artificial chromosome N13R10 was determined. Comparison to pathogenic salivary gland-derived virus revealed 13 differences, 1 of which disrupted overlapping open reading frames encoding GP129 and GP130. Attenuation of N13R10 may arise from an inability to express GP129 and/or GP130.

## GENOME ANNOUNCEMENT

Guinea pig cytomegalovirus (GPCMV) is a model for congenital CMV infection, an important cause of disability in newborns (1). Salivary gland (SG) extracts of strain 22122 (ATCC VR-682) (2) are highly pathogenic *in vivo*. As little as 1.7 × 10^5^ PFU results in 60% dam mortality, and a dose as low as 3.5 × 10^3^ can produce up to 80% pup mortality (3). In this dose range, maternal viremia is common and placental and live-born pup infection rates reach 75% (4). Attenuation occurs upon passage in tissue culture. The genome of a tissue culture-attenuated virus has been cloned as bacterial artificial chromosome (BAC) N13R10 (5). Although N13R10-derived virus grows like the wild type in cell culture (5), it is highly attenuated *in vivo*. A high dose (10^8^ PFU) given to young female guinea pigs caused transient viremia and delayed weight gain, but no mortality (6). A dose of 5 × 10^5^ PFU caused congenital infections in 60% of live-born pups but relatively low (17%) pup mortality (7). Thus, compared to SG virus, adult guinea pigs tolerate a 5,880-fold higher dose of N13R10 without significant illness or mortality, while a 140-fold higher dose of N13R10 during pregnancy produces lower rates of fetal transmission and pup loss.

The sequence of pathogenic SG-22122 (KC503762) was recently obtained by direct sequencing of DNA extracted from SG homogenate (8). To identify potential attenuating mutations, the N13R10 BAC was sequenced on a 454 GS Junior sequencer (Roche). A total of 176,995 reads (average length 405 bp) were *de novo* assembled using CLC Genomics Workbench version 7.0.4 (CLC bio). Contigs were oriented by BLAST alignment with reference sequence KC503762, and then joined manually. Joined contigs were compared to reference sequence KC503762 and all gaps and differences were resolved by targeted Sanger sequencing (GENEWIZ, Inc., South Plainfield, NJ).

Thirteen differences were observed between N13R10 and KC503762: seven 1-bp differences in the lengths of homopolymeric tracts, two 3-bp differences in the lengths of 3-bp repeats, one 1-bp and one 4-bp deletion, and two 1-bp substitutions. Only two differences altered currently annotated protein coding sequences: (i) a 4-bp deletion/frameshift affecting overlapping genes encoding GP129 and GP130 and (ii) a single amino acid substitution in GP130 that is 3′ of the 4-bp deletion/frameshift.

The function of GP130 is unknown. GP129 is part of a complex analogous to one that mediates entry of human cytomegalovirus into monocytes and epithelial and endothelial cells (9–12). In GPCMV, mutations disrupting this complex have been hypothesized to contribute to attenuation (9, 12–14). The absence of other mutations impacting annotated coding sequences in the N13R10 genome strengthens this hypothesis and further suggests that loss of GP129 and/or GP130 may be the primary and perhaps only mechanism of N13R10’s attenuation. If so, genetic repair of N13R10 to express wild-type GP129 and GP130 may result in a highly pathogenic BAC-cloned GPCMV. The ability to construct viral mutants in a viral strain with enhanced pathogenicity would greatly facilitate vaccine and pathogenesis studies in the GPCMV model of congenital infection.

### Nucleotide sequence accession number.

The annotated N13R10 sequence has been deposited in GenBank with the accession number KM384022.
